# *catena*-Poly[[aqua­trimethyl­tin(IV)]-{[trimethyl­tin(IV)]-μ_3_-thio­phene-2,5-dicarboxyl­ato}]

**DOI:** 10.1107/S1600536808043614

**Published:** 2009-01-08

**Authors:** Sheng-Xiang Yang, Yue-Zhong Li, Kun Jiang

**Affiliations:** a Huai Nan Union University, Huainan, Anhui 232038, People’s Republic of China

## Abstract

In the title compound, [Sn_2_(CH_3_)_6_(C_6_H_2_O_4_S)(H_2_O)]_*n*_, each of the two crystallographically independent Sn atoms exhibits a distorted trigonal–bipyramidal coordination geometry formed by two O and three C atoms. The coordinated water mol­ecule plays an important role in crystal packing consolidation *via* O—H⋯O hydrogen bonding.

## Related literature

For related structures, see: Prabusankar & Murugavel (2004 ▶); Bhandari *et al.* (1998 ▶); Ma *et al.* (2006 ▶). 
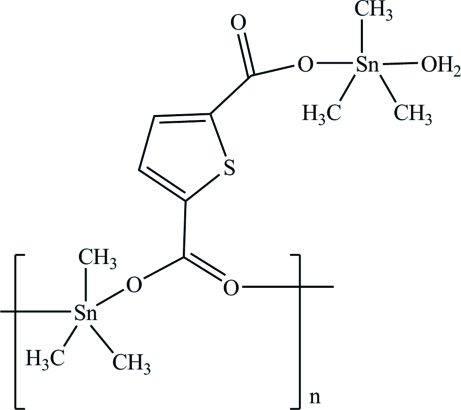


## Experimental

### 

#### Crystal data

[Sn_2_(CH_3_)_6_(C_6_H_2_O_4_S)(H_2_O)]
*M* *_r_* = 515.74
Monoclinic, 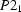

*a* = 7.2761 (16) Å
*b* = 10.467 (2) Å
*c* = 12.894 (3) Å
β = 102.768 (2)°
*V* = 957.7 (4) Å^3^
*Z* = 2
Mo *K*α radiation
μ = 2.73 mm^−1^
*T* = 298 (2) K
0.40 × 0.30 × 0.22 mm

#### Data collection

Bruker SMART CCD area-detector diffractometer
Absorption correction: multi-scan (*SADABS*; Sheldrick, 1996 ▶) *T*_min_ = 0.408, *T*_max_ = 0.585 (expected range = 0.383–0.549)
4866 measured reflections
3204 independent reflections
2950 reflections with *I* > 2σ(*I*)
*R*_int_ = 0.024

#### Refinement

*R*[*F* ^2^ > 2σ(*F* ^2^)] = 0.028
*wR*(*F* ^2^) = 0.067
*S* = 1.03
3204 reflections
193 parameters
1 restraint
H atoms treated by a mixture of independent and constrained refinement
Δρ_max_ = 0.40 e Å^−3^
Δρ_min_ = −0.41 e Å^−3^
Absolute structure: Flack (1983 ▶), 1631 Friedel pairs
Flack parameter: −0.06 (3)

### 

Data collection: *SMART* (Siemens, 1996 ▶); cell refinement: *SAINT* (Siemens, 1996 ▶); data reduction: *SAINT*; program(s) used to solve structure: *SHELXS97* (Sheldrick, 2008 ▶); program(s) used to refine structure: *SHELXL97* (Sheldrick, 2008 ▶); molecular graphics: *SHELXTL* (Sheldrick, 2008 ▶); software used to prepare material for publication: *SHELXTL*.

## Supplementary Material

Crystal structure: contains datablocks I, global. DOI: 10.1107/S1600536808043614/cv2496sup1.cif

Structure factors: contains datablocks I. DOI: 10.1107/S1600536808043614/cv2496Isup2.hkl

Additional supplementary materials:  crystallographic information; 3D view; checkCIF report

## Figures and Tables

**Table 1:** Selected bond lengths (Å)

Sn1—C9	2.114 (7)
Sn1—C7	2.117 (7)
Sn1—C8	2.120 (8)
Sn1—O1	2.135 (5)
Sn1—O2^i^	2.641 (5)
Sn2—C10	2.108 (7)
Sn2—C12	2.108 (7)
Sn2—C11	2.114 (7)
Sn2—O3	2.178 (4)
Sn2—O5	2.495 (5)

Symmetry code: (i) 
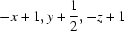
.

**Table 2:** Hydrogen-bond geometry (Å, °)

*D*—H⋯*A*	*D*—H	H⋯*A*	*D*⋯*A*	*D*—H⋯*A*
O5—H1⋯O3^ii^	0.85 (8)	2.38 (8)	3.050 (7)	136 (7)
O5—H2⋯O4^iii^	0.85 (7)	1.98 (8)	2.765 (6)	153 (7)

Symmetry codes: (ii) 
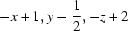
; (iii) 
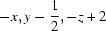
.

**Fig. 1.**
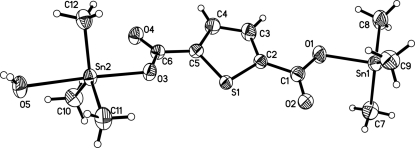
A portion of the polymeric chain of the title compound showing the atomic numbering and 30% probability displacement ellipsoids.

**Fig. 2.**
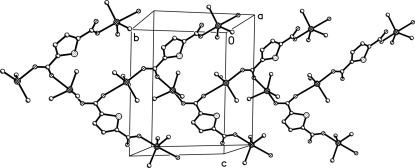
One-dimensional polymeric chain in the title compound.

## supplementary crystallographic information

### Comment

Organotin complexes are attracting more and more attention because of their considerable structural diversity and interesting topologies (Prabusankar *et al.*, 2004). From the coordinated viewpoint, those dicarboxylate ligands with additional donor atoms, has been revealed to help the construction of interesting topologies (Bhandari *et al.*, 1998). Herein, we report the structure of the title complex, (I).

The title compound (Fig.1) forms an extended one-dimensional chain structure along the *b* axis arising from Sn—O bridges to ligands. The Sn1 atom has distorted trigonal-bipyramidal geometry, with atoms O1 and O2 in axial positions [O1—Sn1—O2(1 - *x*, *y* + 1/2, 1 - *z*) = 172.06 (17) °] and the C atoms of the three methyl groups in equatorial positions. Associated with the sum of the angles subtended at the Sn1 in the equatorial plane is 357.9 (4) °, indicating approximate coplanarity for these atoms; and the Sn1—O1 distance 2.135 (5) Å and Sn1—O2^i^ distance 2.641 (5) Å (Table 1), are close to the reported values for organotin compounds (Ma *et al.*, 2006). The environment of the Sn2 atom is approximate to Sn1.

### Experimental

The reaction was carried out under nitrogen atmosphere. Thiophenn-2,5-dicarboxylic acid (1 mmol) and sodium ethoxide (2 mmol) were added to a stirred solution of benzene (30 ml) in a Schlenk flask and stirred for 0.5 h. Trimethyltin chloride (2 mmol) was then added to the reactor and the reaction mixture was stirred for 12 h at room temperature. The resulting clear solution was evaporated under vacuum. The product was crystallized from dichloromethane to yield colourless blocks of compound (yield 82%. m.p.463k). Anal. Calcd (%) for C_12_H_22_O_5_SSn_2_ (Mr = 515.74): C, 27.94; H, 4.30; Found (%): C, 27.65; H, 4.57.

### Refinement

C-bound H atoms were geometrically positioned [C–H 0.93–0.96 Å] and treated as riding, with *U*_iso_(H) = 1.2–1.5*U*_eq_(C). Atoms H1 and H2 were located on a difference Fourier map and refined with bond restraints O–H=0.85 (7) Å and constrained *U*_iso_(H) = 1.1*U*_eq_(O).

### Crystal data

**Table:**

[Sn_2_(CH_3_)_6_(C_6_H_2_O_4_S)(H_2_O)]	*F*(000) = 500
*M**_r_* = 515.74	*D*_x_ = 1.788 Mg m^−^^3^
Monoclinic, *P*2_1_	Mo *K*α radiation, λ = 0.71073 Å
*a* = 7.2761 (16) Å	Cell parameters from 3167 reflections
*b* = 10.467 (2) Å	θ = 2.5–27.3°
*c* = 12.894 (3) Å	µ = 2.73 mm^−^^1^
β = 102.768 (2)°	*T* = 298 K
*V* = 957.7 (4) Å^3^	Block, colourless
*Z* = 2	0.40 × 0.30 × 0.22 mm

### Data collection

**Table:**

Bruker SMART CCD area-detector diffractometer	3204 independent reflections
Radiation source: fine-focus sealed tube	2950 reflections with *I* > 2σ(*I*)
graphite	*R*_int_ = 0.024
φ and ω scans	θ_max_ = 25.0°, θ_min_ = 1.6°
Absorption correction: multi-scan (*SADABS*; Sheldrick, 1996)	*h* = −7→8
*T*_min_ = 0.408, *T*_max_ = 0.585	*k* = −12→12
4866 measured reflections	*l* = −14→15

### Refinement

**Table:**

Refinement on *F*^2^	Secondary atom site location: difference Fourier map
Least-squares matrix: full	Hydrogen site location: inferred from neighbouring sites
*R*[*F*^2^ > 2σ(*F*^2^)] = 0.028	H atoms treated by a mixture of independent and constrained refinement
*wR*(*F*^2^) = 0.067	*w* = 1/[σ^2^(*F*_o_^2^) + (0.0298*P*)^2^ + 0.4614*P*] where *P* = (*F*_o_^2^ + 2*F*_c_^2^)/3
*S* = 1.03	(Δ/σ)_max_ = 0.001
3204 reflections	Δρ_max_ = 0.40 e Å^−^^3^
193 parameters	Δρ_min_ = −0.41 e Å^−^^3^
1 restraint	Absolute structure: Flack (1983), 1631 Friedel pairs
Primary atom site location: structure-invariant direct methods	Flack parameter: −0.06 (3)

### Special details

**Table:**

Geometry. All e.s.d.'s (except the e.s.d. in the dihedral angle between two l.s. planes) are estimated using the full covariance matrix. The cell e.s.d.'s are taken into account individually in the estimation of e.s.d.'s in distances, angles and torsion angles; correlations between e.s.d.'s in cell parameters are only used when they are defined by crystal symmetry. An approximate (isotropic) treatment of cell e.s.d.'s is used for estimating e.s.d.'s involving l.s. planes.
Refinement. Refinement of *F*^2^ against ALL reflections. The weighted *R*-factor *wR* and goodness of fit *S* are based on *F*^2^, conventional *R*-factors *R* are based on *F*, with *F* set to zero for negative *F*^2^. The threshold expression of *F*^2^ > σ(*F*^2^) is used only for calculating *R*-factors(gt) *etc*. and is not relevant to the choice of reflections for refinement. *R*-factors based on *F*^2^ are statistically about twice as large as those based on *F*, and *R*- factors based on ALL data will be even larger.

### Fractional atomic coordinates and isotropic or equivalent isotropic displacement parameters (Å^2^)

**Table:**

	*x*	*y*	*z*	*U*_iso_*/*U*_eq_	
Sn1	0.40954 (6)	0.66275 (4)	0.52089 (3)	0.05596 (13)	
Sn2	0.29616 (5)	−0.21300 (4)	0.94966 (3)	0.04704 (11)	
O1	0.2935 (8)	0.5249 (5)	0.6105 (4)	0.0697 (13)	
O2	0.4490 (8)	0.3555 (5)	0.5684 (4)	0.0712 (13)	
O3	0.2928 (6)	−0.0330 (4)	0.8646 (3)	0.0549 (11)	
O4	0.0302 (6)	0.0281 (5)	0.9120 (3)	0.0620 (12)	
O5	0.3171 (8)	−0.4236 (5)	1.0422 (5)	0.0697 (14)	
S1	0.3024 (2)	0.17295 (16)	0.71215 (11)	0.0493 (3)	
C1	0.3378 (10)	0.4063 (7)	0.6153 (5)	0.0571 (16)	
C2	0.2486 (8)	0.3306 (5)	0.6871 (4)	0.0463 (14)	
C3	0.1297 (10)	0.3741 (7)	0.7489 (6)	0.0668 (19)	
H3	0.0850	0.4575	0.7468	0.080*	
C4	0.0833 (9)	0.2787 (8)	0.8154 (5)	0.0638 (16)	
H4	0.0038	0.2919	0.8618	0.077*	
C5	0.1680 (8)	0.1643 (7)	0.8045 (4)	0.0463 (12)	
C6	0.1558 (8)	0.0462 (6)	0.8644 (4)	0.0476 (14)	
C7	0.2903 (11)	0.5827 (9)	0.3703 (5)	0.079 (2)	
H7A	0.3624	0.5094	0.3586	0.118*	
H7B	0.2918	0.6451	0.3159	0.118*	
H7C	0.1627	0.5574	0.3681	0.118*	
C8	0.2492 (14)	0.8103 (8)	0.5708 (8)	0.097 (3)	
H8A	0.1767	0.7757	0.6181	0.145*	
H8B	0.1657	0.8470	0.5099	0.145*	
H8C	0.3324	0.8752	0.6072	0.145*	
C9	0.6959 (10)	0.6383 (9)	0.5970 (6)	0.080 (2)	
H9A	0.7046	0.5871	0.6597	0.119*	
H9B	0.7523	0.7202	0.6166	0.119*	
H9C	0.7610	0.5964	0.5495	0.119*	
C10	0.0317 (9)	−0.2808 (8)	0.8666 (6)	0.0686 (19)	
H10A	−0.0630	−0.2177	0.8689	0.103*	
H10B	0.0016	−0.3582	0.8991	0.103*	
H10C	0.0361	−0.2974	0.7940	0.103*	
C11	0.5359 (11)	−0.2714 (8)	0.8945 (7)	0.084 (3)	
H11A	0.4987	−0.3313	0.8374	0.125*	
H11B	0.6248	−0.3110	0.9516	0.125*	
H11C	0.5931	−0.1982	0.8696	0.125*	
C12	0.3463 (10)	−0.1258 (7)	1.1007 (5)	0.0656 (19)	
H12A	0.2520	−0.0619	1.1015	0.098*	
H12B	0.4686	−0.0867	1.1156	0.098*	
H12C	0.3411	−0.1892	1.1538	0.098*	
H1	0.405 (12)	−0.434 (8)	1.097 (6)	0.079*	
H2	0.233 (11)	−0.442 (8)	1.076 (6)	0.079*	

### Atomic displacement parameters (Å^2^)

**Table:**

	*U*^11^	*U*^22^	*U*^33^	*U*^12^	*U*^13^	*U*^23^
Sn1	0.0634 (3)	0.0477 (2)	0.0566 (2)	0.0046 (2)	0.0130 (2)	0.0066 (2)
Sn2	0.03417 (18)	0.0448 (2)	0.0646 (2)	0.00064 (19)	0.01612 (16)	0.0085 (2)
O1	0.092 (4)	0.042 (3)	0.081 (3)	0.010 (3)	0.033 (3)	0.019 (2)
O2	0.099 (4)	0.052 (3)	0.073 (3)	0.003 (3)	0.041 (3)	0.002 (2)
O3	0.051 (2)	0.050 (2)	0.070 (3)	0.013 (2)	0.026 (2)	0.018 (2)
O4	0.051 (3)	0.066 (3)	0.075 (3)	0.009 (2)	0.028 (2)	0.014 (2)
O5	0.057 (3)	0.059 (3)	0.099 (4)	−0.003 (2)	0.029 (3)	0.023 (3)
S1	0.0592 (9)	0.0388 (8)	0.0543 (7)	0.0041 (7)	0.0223 (7)	0.0034 (7)
C1	0.065 (4)	0.054 (4)	0.050 (3)	0.002 (3)	0.008 (3)	0.007 (3)
C2	0.049 (3)	0.041 (3)	0.047 (3)	0.005 (2)	0.007 (3)	0.006 (2)
C3	0.069 (5)	0.047 (4)	0.087 (5)	0.019 (3)	0.022 (4)	0.016 (3)
C4	0.066 (4)	0.055 (4)	0.078 (4)	0.016 (4)	0.034 (3)	0.008 (4)
C5	0.042 (3)	0.049 (3)	0.049 (3)	0.007 (3)	0.011 (2)	0.002 (3)
C6	0.040 (3)	0.051 (4)	0.053 (3)	0.004 (3)	0.015 (3)	0.003 (3)
C7	0.067 (5)	0.098 (7)	0.064 (4)	−0.020 (4)	0.000 (4)	0.004 (4)
C8	0.125 (8)	0.051 (5)	0.135 (8)	0.011 (5)	0.073 (7)	0.012 (5)
C9	0.070 (5)	0.086 (6)	0.073 (4)	−0.002 (4)	−0.003 (4)	−0.002 (4)
C10	0.047 (4)	0.075 (5)	0.080 (4)	−0.008 (3)	0.004 (3)	−0.004 (4)
C11	0.071 (5)	0.064 (5)	0.133 (7)	0.024 (4)	0.059 (5)	0.025 (5)
C12	0.060 (4)	0.059 (4)	0.071 (4)	−0.005 (3)	−0.001 (3)	0.011 (3)

### Geometric parameters (Å, °)

**Table:**

Sn1—C9	2.114 (7)	C4—C5	1.368 (10)
Sn1—C7	2.117 (7)	C4—H4	0.9300
Sn1—C8	2.120 (8)	C5—C6	1.470 (9)
Sn1—O1	2.135 (5)	C7—H7A	0.9600
Sn1—O2^i^	2.641 (5)	C7—H7B	0.9600
Sn2—C10	2.108 (7)	C7—H7C	0.9600
Sn2—C12	2.108 (7)	C8—H8A	0.9600
Sn2—C11	2.114 (7)	C8—H8B	0.9600
Sn2—O3	2.178 (4)	C8—H8C	0.9600
Sn2—O5	2.495 (5)	C9—H9A	0.9600
O1—C1	1.282 (9)	C9—H9B	0.9600
O2—C1	1.233 (8)	C9—H9C	0.9600
O3—C6	1.296 (7)	C10—H10A	0.9600
O4—C6	1.223 (6)	C10—H10B	0.9600
O5—H1	0.85 (8)	C10—H10C	0.9600
O5—H2	0.85 (7)	C11—H11A	0.9600
S1—C5	1.702 (5)	C11—H11B	0.9600
S1—C2	1.711 (6)	C11—H11C	0.9600
C1—C2	1.474 (9)	C12—H12A	0.9600
C2—C3	1.376 (8)	C12—H12B	0.9600
C3—C4	1.405 (10)	C12—H12C	0.9600
C3—H3	0.9300		
			
C9—Sn1—C7	122.7 (3)	C6—C5—S1	121.5 (5)
C9—Sn1—C8	120.0 (4)	O4—C6—O3	124.0 (6)
C7—Sn1—C8	115.2 (4)	O4—C6—C5	122.2 (6)
C9—Sn1—O1	97.7 (3)	O3—C6—C5	113.8 (5)
C7—Sn1—O1	95.6 (3)	Sn1—C7—H7A	109.5
C8—Sn1—O1	91.0 (2)	Sn1—C7—H7B	109.5
C9—Sn1—O2^i^	81.7 (3)	H7A—C7—H7B	109.5
C7—Sn1—O2^i^	91.3 (2)	Sn1—C7—H7C	109.5
C8—Sn1—O2^i^	82.5 (3)	H7A—C7—H7C	109.5
O1—Sn1—O2^i^	172.06 (17)	H7B—C7—H7C	109.5
C10—Sn2—C12	124.6 (3)	Sn1—C8—H8A	109.5
C10—Sn2—C11	117.4 (4)	Sn1—C8—H8B	109.5
C12—Sn2—C11	116.7 (3)	H8A—C8—H8B	109.5
C10—Sn2—O3	97.4 (3)	Sn1—C8—H8C	109.5
C12—Sn2—O3	94.1 (2)	H8A—C8—H8C	109.5
C11—Sn2—O3	89.9 (2)	H8B—C8—H8C	109.5
C10—Sn2—O5	84.1 (3)	Sn1—C9—H9A	109.5
C12—Sn2—O5	87.8 (2)	Sn1—C9—H9B	109.5
C11—Sn2—O5	86.4 (2)	H9A—C9—H9B	109.5
O3—Sn2—O5	176.36 (17)	Sn1—C9—H9C	109.5
C1—O1—Sn1	123.8 (4)	H9A—C9—H9C	109.5
C6—O3—Sn2	118.5 (4)	H9B—C9—H9C	109.5
Sn2—O5—H1	118 (6)	Sn2—C10—H10A	109.5
Sn2—O5—H2	118 (6)	Sn2—C10—H10B	109.5
H1—O5—H2	92 (7)	H10A—C10—H10B	109.5
C5—S1—C2	92.3 (3)	Sn2—C10—H10C	109.5
O2—C1—O1	125.4 (6)	H10A—C10—H10C	109.5
O2—C1—C2	120.3 (6)	H10B—C10—H10C	109.5
O1—C1—C2	114.2 (6)	Sn2—C11—H11A	109.5
C3—C2—C1	127.4 (6)	Sn2—C11—H11B	109.5
C3—C2—S1	110.9 (4)	H11A—C11—H11B	109.5
C1—C2—S1	121.5 (5)	Sn2—C11—H11C	109.5
C2—C3—C4	112.6 (6)	H11A—C11—H11C	109.5
C2—C3—H3	123.7	H11B—C11—H11C	109.5
C4—C3—H3	123.7	Sn2—C12—H12A	109.5
C5—C4—C3	112.7 (6)	Sn2—C12—H12B	109.5
C5—C4—H4	123.7	H12A—C12—H12B	109.5
C3—C4—H4	123.7	Sn2—C12—H12C	109.5
C4—C5—C6	126.9 (5)	H12A—C12—H12C	109.5
C4—C5—S1	111.5 (5)	H12B—C12—H12C	109.5

Symmetry codes: (i) −*x*+1, *y*+1/2, −*z*+1.

### Hydrogen-bond geometry (Å, °)

**Table:**

*D*—H···*A*	*D*—H	H···*A*	*D*···*A*	*D*—H···*A*
O5—H1···O3^ii^	0.85 (8)	2.38 (8)	3.050 (7)	136 (7)
O5—H2···O4^iii^	0.85 (7)	1.98 (8)	2.765 (6)	153 (7)

Symmetry codes: (ii) −*x*+1, *y*−1/2, −*z*+2; (iii) −*x*, *y*−1/2, −*z*+2.
